# Community Champions: The Crucial Contribution of an Independent Pharmacy in COVID-19 Vaccination Efforts in an Underserved Community

**DOI:** 10.3390/ijerph20196881

**Published:** 2023-10-03

**Authors:** Chardaé Whitner, Stacey D. Curtis, John M. Allen, Kevin J. Duane

**Affiliations:** 1 Department of Pharmacotherapy and Translation Research, Jacksonville Campus, College of Pharmacy, University of Florida, 580 W. 8th Street, Jacksonville, FL 32209, USA; 2 Department of Pharmacotherapy and Translation Research, Gainesville Campus, College of Pharmacy, University of Florida, 1225 Center Drive, Gainesville, FL 32610, USA; scurtis@cop.ufl.edu; 3 Department of Pharmacotherapy and Translation Research, Orlando Campus, College of Pharmacy, University of Florida, 6550 Sanger Rd., Orlando, FL 32827, USA; john.allen@cop.ufl.edu; 4 Panama Pharmacy, 7307 N. Main St, Jacksonville, FL 32208, USA; kevin@panamarx.com

**Keywords:** independent pharmacy, health disparities, underserved communities, COVID-19 vaccination, healthcare delivery

## Abstract

Historically, pharmacists in independent community pharmacies have been pivotal in promoting community health. During the COVID-19 pandemic, they demonstrated their commitment by advocating for vaccination and providing accessible care, particularly in underserved communities. By addressing disparities, implementing strategies like mobile clinics and community outreach, and fostering trust and engagement, independent community pharmacists played a crucial role in bridging gaps in healthcare access for vulnerable populations and mitigating the impact of the COVID-19 pandemic.

## 1. Introduction

Historically, independent community pharmacies have played a vital role in bolstering community health by offering a diverse spectrum of healthcare services. The responsibilities of apothecaries and pharmacists have spanned a wide range, encompassing the provision of natural and herbal remedies, compounding pharmaceuticals, and disseminating education on preventative measures to combat the transmission of diseases. Notably, during the 1920s Spanish influenza pandemic, pharmacists emerged as indispensable frontline professionals, assuming a pivotal role as primary sources of essential healthcare support [1].

Recent years have witnessed pharmacists continuing to fulfill this crucial role, particularly during the ongoing COVID-19 pandemic. The unwavering commitment to supporting their communities during these unprecedented times underscores their status as enduring pillars of community health. Independent community pharmacists and pharmacy owners have demonstrated remarkable dedication by taking a resolute stance amid the challenges posed by the COVID-19 pandemic. Their concerted efforts ensure the delivery of optimal and accessible healthcare services to all community members, with a particular focus on those residing in underserved and low socio-economic communities [2].

This manuscript is dedicated to an in-depth exploration of the COVID-19 vaccination advocacy practices employed by pharmacists practicing at an urban-based independent community pharmacy site in Jacksonville, FL. Their diverse team of pharmacists reflects the expansive demographics of the community itself, which is known for its ethnic diversity. Jacksonville, FL, is a city characterized by its diverse population, with residents hailing from various racial, ethnic, and socio-economic backgrounds. According to recent census data, the community centered around the pharmacy represents a diverse ethnic tapestry, with approximately 90% identifying as African American or Black, 4% as non-Hispanic White, 3% as Hispanic or Latino, and 3% representing various other ethnicities [3]. The community’s demographics underscore the importance of equitable vaccine distribution, particularly in reaching underserved and minority communities disproportionately affected by COVID-19.

The dedicated pharmacy staff, mirroring the diversity of Jacksonville, FL, consists of team members from different racial and ethnic backgrounds, including two non-Hispanic White and two African American pharmacists. Additionally, the pharmacy technician group comprises three African Americans, three non-Hispanic White individuals, and one Asian American. The diversity within the pharmacy team aligns with the broader goal of addressing vaccine hesitancy and fostering trust within the diverse community they serve.

This pharmacy team administered a substantial number of COVID-19 vaccines to community members. Over the span of five months, they successfully administered over 4000 COVID-19 vaccine doses during the initial availability of the vaccine. This significant achievement highlights their commitment to ensuring that a large segment of the population, including those in underserved communities, had access to and received the much-needed COVID-19 vaccine.

The intersection of a diverse pharmacy team, a culturally rich city, and a substantial number of vaccines administered forms the backdrop against which we explore the multifaceted strategies employed to advocate for COVID-19 vaccination in a low socio-economic community that historically has been underserved. The team at Panama Pharmacy has become community champions, fostering mutually respectful connections within the community by serving their patients in a manner that is equitable, accessible, and sensitive to their unique needs.

## 2. Accessibility

According to the literature, there was a significant disparity in COVID-19 vaccination rates between individuals residing in socially vulnerable areas and those in well-resourced areas, even though the former were disproportionately affected by the virus [4]. This disparity is often attributed to vaccine hesitancy, but other crucial factors, such as limited accessibility to vaccination sites, also played a significant role [5]. To address this issue, an independent community pharmacy took proactive steps. The independent community pharmacy became one of the first sites in the state of Florida to offer the COVID-19 vaccine to the community. By doing so, they provided earlier access and offered close proximity for community members to receive the much-needed vaccine.

However, disparities in vaccination distribution also emerged due to information technology (IT) barriers. Numerous retail community pharmacies implemented electronic registration systems for vaccine appointments to enhance efficiency. Unfortunately, this approach presented challenges for individuals without internet access or limited digital literacy skills, a common issue recognized among elderly populations and those in lower socio-economic areas [6].

To address this issue, the pharmacy took proactive measures by allowing individuals facing difficulties accessing the online registration form to visit the pharmacy site and register for the vaccine with the assistance of pharmacy staff members. Moreover, walk-in appointments were made available to individuals who lacked the necessary resources to reserve an appointment for the COVID-19 vaccination from their homes. These efforts aimed to ensure equitable access to the vaccine and accommodate individuals who faced IT access and proficiency barriers.

Another crucial aspect of accessibility observed by the pharmacists at this independent pharmacy was the limited access to transportation for certain patients. Recognizing this challenge, pharmacy practitioners took the initiative and established mobile vaccine clinics to reach individuals who faced barriers related to transportation. These clinics were particularly focused on providing early access to the COVID-19 vaccine for individuals aged 65 and older, as prioritizing the elderly members of the community was an early initiative. Examples of sites visited by pharmacists included long-term care centers and local apartment complexes. By implementing mobile vaccine clinics, the pharmacy aimed to ensure that individuals who lacked transportation resources could still receive timely access to the vaccination (Table 1).

Despite the proactive efforts of the independent pharmacy to address barriers to vaccine access, there were additional challenges encountered in the process. One such challenge was the delayed recognition and support from the Centers for Medicare & Medicaid Services (CMS) regarding vaccines administered outside of the clinical setting. While the pharmacy had already established mobile vaccine clinics and provided vaccines to vulnerable populations, including elderly individuals, CMS took several months to provide payment and support for these services. CMS’s delayed response created additional financial strain on the pharmacy and hindered its ability to expand its efforts broadly. Despite the obstacles faced, the pharmacy remained committed to serving the community and ensuring equitable access to the COVID-19 vaccine [7].

## 3. Diversification and Reinvention

The recognition of mobile vaccine clinic importance grew alongside the increasing availability of COVID-19 vaccines. Pharmacists understood the necessity of ensuring vaccine accessibility beyond individuals’ homes. In response, community outreach initiatives were established, bringing the mobile vaccine clinic directly to people in their communities and workplaces. This approach specifically aimed to overcome the challenges faced by essential workers, who often had demanding schedules and limited time to visit vaccination sites. According to Georgetown Law, essential workers are defined as an individual whose work is centered around operations and services that are critical to maintaining the core functions of public health, society, and the economy and whose work does not offer the ability to work remotely [8]. Essential workers, found in sectors such as health facilities, farms, factories, food production and processing, grocery stores, and public transportation, were frequently from racial and ethnic minority groups and underserved communities. Their close contact with coworkers and the public puts them at higher risk of COVID-19 exposure [9]. The implementation of mobile vaccine clinics and community outreach efforts was crucial in ensuring equitable access to COVID-19 vaccines for those who were working to serve others.

To facilitate accessibility for essential workers, the pharmacists proactively connected with the leadership of organizations in the local community. Collaboratively, they established mobile vaccine clinics at various locations, allowing workers to receive vaccinations during their working hours. These locations included the local zoo, local schools, restaurants and bars, a food packing plant, and a shipyard. By offering vaccinations at these sites, the pharmacy aimed to ensure that essential workers could access the vaccine conveniently and without additional disruptions to their work schedules.

This proactive approach not only aimed to address the challenges faced by essential workers but also emphasized the importance of reaching out to different segments of the community in their own familiar settings. By bringing the vaccine directly to these locations, the pharmacy sought to increase accessibility and promote equitable distribution, ultimately contributing to the broader goal of mitigating the impact of COVID-19 in the community (Table 2).

## 4. Community Engagement and Personalized Care

In addition to actively addressing the inequitable distribution of COVID-19 vaccines, pharmacists at this site also recognized the importance of adopting a communal approach to ensure vaccine access for all community members. As mentioned earlier, vaccine hesitancy is a significant barrier among socially vulnerable populations. This hesitancy stems from factors such as historical trauma, mistrust, limited patient education about vaccines, and lack of patient concordance [10].

Acknowledging the value of having racial and ethnic minority pharmacists as part of the team, the pharmacists implemented initiatives to build trust, educate patients, and foster patient-provider relationships. They understood the significance of addressing historical traumas and the need to establish open lines of communication with patients. The pharmacy team actively engaged with the community through education, providing accurate and culturally sensitive information about vaccines. They collaborated with local organizations and trusted individuals to deliver messages promoting the importance of vaccination.

Moreover, the pharmacists emphasized patient concordance by taking the time to listen to patients’ concerns, answer their questions, and address their specific needs. This personalized approach aimed to build trust, enhance understanding, and empower patients to make informed decisions about vaccination. It is worth noting that the current literature suggests that medical care provided by a racially diverse care team greatly increases the likelihood of positive medical outcomes for all patients, particularly for marginalized patients [11] (Table 3).

By implementing these initiatives and leveraging the diverse perspectives within their team, the pharmacists demonstrated a strong commitment to addressing the underlying factors contributing to vaccine hesitancy within socially vulnerable populations. Their efforts yielded promising results, as within the first five months of vaccine availability, 42% of the vaccines administered by the pharmacists were received by patients who self-identified as a racial or ethnic minority. This inclusive and communal approach aimed to bridge the gap in vaccine access, promote trust, and ensure that all community members, regardless of their background, have equitable opportunities to receive COVID-19 vaccines. Through their proactive measures and dedication to the community’s well-being, the pharmacists set an inspiring example for promoting health equity in vaccination efforts.

## 5. Discussion

While the current body of literature is somewhat limited in its scope, exploring the roles and responsibilities, particularly of independent community pharmacists, two previous studies showed that independent community pharmacies were prepared to provide COVID-19 vaccinations to their communities, increasing access for their community’s health needs [2,12]. The pharmacists who operate within the independent community pharmacies possess a unique and special relationship with the population they serve, which cultivates a deep sense of trust between the pharmacist and the patients they serve.

There has been a growing exploration within the literature regarding the importance of trustworthy relationships with community stakeholders and the potential benefits of partnerships with independent community pharmacies. Recognizing the pivotal role these pharmacists who work in these pharmacies play in the healthcare system, researchers have begun to investigate the impact of collaborative efforts between pharmacists and other community stakeholders.

One notable area of study involves leveraging faith-based organizations to increase vaccination rates among non-Hispanic Black and Hispanic/Latino communities. Pharmacists have recognized the influence and reach of faith-based organizations within these communities and have sought to harness these connections to promote vaccination efforts. By partnering with faith-based organizations, pharmacists can engage with community members, provide education about vaccines, address concerns, and ultimately increase vaccination rates within these underserved populations [13].

These initiatives not only highlight the unique position of pharmacists but also underscore the importance of collaboration and innovative approaches in improving public health outcomes. By working together with other trusted members of the community and utilizing community resources such as faith-based organizations, pharmacists can have a significant impact on increasing vaccination rates and promoting better health outcomes for all members of the community, particularly those in marginalized populations.

While the described practices of pharmacists at an urban-based independent community pharmacy in Jacksonville, FL, have been commendable in addressing disparities and promoting community health during the COVID-19 pandemic, there are several avenues for future research to further advance the field of independent community pharmacy. Studies can be conducted examining the feasibility and effectiveness of replicating the practices implemented by the independent pharmacy in other urban, suburban, and rural settings. Understanding the scalability and adaptability of these strategies can help guide future implementation efforts and promote widespread adoption across diverse communities.

Additionally, investigating the long-term impact of the efforts undertaken by independent community pharmacies on vaccination rates and health outcomes in underserved communities is important. Longitudinal studies can help assess the sustainability of these initiatives and their influence on improving overall community health beyond the immediate crisis. By pursuing these research avenues, the field of independent community pharmacy can further advance evidence-based practices and develop innovative strategies to improve health outcomes and promote health equity in underserved communities.

## 6. Conclusions

The dedicated efforts of pharmacists working in independent community pharmacies located in underserved communities were instrumental in advocating for COVID-19 vaccination and ensuring optimal and accessible care for their communities. These healthcare professionals took a resolute stance and implemented proactive strategies to address disparities, promote accessibility, and foster community engagement. By doing so, they demonstrated their unwavering commitment to community health during those unprecedented times.

Through the pharmacists’ diligent work, independent community pharmacies played a pivotal role in mitigating the impact of the COVID-19 pandemic and safeguarding the well-being of community members. Their efforts went beyond simply administering vaccines; they actively worked towards addressing the unique challenges faced by underserved communities. By focusing on areas with limited resources and higher vulnerability, these pharmacists made a significant impact in bridging gaps in healthcare access and reducing disparities.

The dedication of pharmacists in independent community pharmacies in advocating for COVID-19 vaccination in underserved communities serves as an inspiring example of community-driven healthcare. Their commitment to providing optimal care, addressing disparities, and engaging with the community not only helped overcome barriers but also built trust and confidence in vaccination. Moving forward, their experiences and practices can serve as valuable lessons and inspire future endeavors to ensure accessible and equitable healthcare for all well-being of their communities. Independent community pharmacies serve as the lifeblood for low socio-economic, underserved, and rural communities becoming community champions of healthcare for those they serve.

## Figures and Tables

**Table 1:** Accessibility Initiatives.

Initiative	Description
Reduction in Information Technology Barriers	Pharmacy staff actively assist individuals with the vaccine registration process. Provided the use of pharmacy-owned resources.
Implementation of Walk-in Appointments	Walk-in appointments are introduced during the initial phases of limited vaccine availability when most vaccine sites required an appointment only
Mobile Vaccine Clinics Implementation	Mobile vaccine clinics are established to provide accessibility to at-risk populations.

**Table 2:** Diversification and Reinvention Initiatives.

Initiative	Description
Establishment of Mobile Vaccine Clinics	Mobile vaccine clinics were introduced to reach underserved communities and essential workers, providing vaccination access beyond traditional healthcare settings.
Community Outreach Initiatives	Pharmacists actively engaged with local organizations and workplaces to bring COVID-19 vaccines directly to people in their communities.
Targeting Essential Workers	Vaccine clinics were set up at essential work sites (e.g., local schools, restaurants, factories) to accommodate workers with demanding schedules.

**Table 3:** Community Engagement and Personalized Care Initiatives.

Initiative	Description
Building Trust and Relationships	Mobile vaccine clinics were introduced to reach underserved communities and essential workers, providing vaccination access beyond traditional healthcare settings.
Increased Vaccination Rates among Marginalized Communities	Proactive measures leading to 42% of vaccines administered within the first five months going to patients who self-identified as a racial or ethnic minority.
Leveraging Racial and Ethnic Diversity	Recognizing the value of racial and ethnic minority pharmacists in the team and leveraging diverse perspectives to address vaccine hesitancy.

## Data Availability

Not applicable.
